# Diagnostic accuracy of fine needle aspiration biopsy for detection of malignancy in pediatric thyroid nodules: protocol for a systematic review and meta-analysis

**DOI:** 10.1186/s13643-015-0109-0

**Published:** 2015-09-24

**Authors:** Sarah W. Lai, Derek J. Roberts, Doreen M. Rabi, Karin Y. Winston

**Affiliations:** Department of Surgery, University of Calgary, Calgary, AB Canada; Department of Gastrointestinal Sciences, University of Calgary, Calgary, AB Canada; Department of Community Health Sciences, University of Calgary, Calgary, AB Canada; Department of Medicine, University of Calgary, Calgary, AB Canada; Department of Pediatrics, University of Calgary, Calgary, AB Canada; Department of Pediatric Surgery, Alberta Children’s Hospital, 2888 Shaganappi Trail NW, Calgary, AB Canada T3B 6A8; Intensive Care Unit Administration, Ground Floor McCaig Tower, Foothills Medical Centre, 3134 Hospital Drive NW, Calgary, AB Canada T2N 5A1

**Keywords:** Fine needle biopsy, Pediatric, Thyroid nodule, Thyroid cancer, Meta-analysis, Systematic review, Diagnostic accuracy, Sensitivity, Specificity, Likelihood ratio

## Abstract

**Background:**

Fine needle aspiration biopsy (FNAB) is an accurate test commonly used to determine whether thyroid nodules are malignant in adults. However, less is known about its diagnostic accuracy for this purpose in children, where conduct of FNAB is less frequent, more technically challenging, and pre-test probabilities of malignancy are often higher. The purpose of this systematic review is to evaluate the diagnostic accuracy of FNAB for the detection of malignancy in pediatric thyroid nodules.

**Methods:**

We will search electronic bibliographic databases (MEDLINE, EMBASE, the Cochrane Library, and Evidence-Based Medicine) from their date of inception, reference lists of included articles, proceedings from relevant conferences, and the table of contents of the Journal of Pediatric Surgery (January 2007–present). Two reviewers will independently screen titles and abstracts and identify diagnostic accuracy studies involving FNAB of the thyroid in children. We will include studies comparing FNAB to a reference standard of surgical histopathology or clinical follow-up for detection of malignancy in pediatric thyroid nodules. Two investigators will independently extract data and assess risk of bias using the Quality of Diagnostic Accuracy Studies-II tool. Pooled estimates of sensitivity, specificity, and positive and negative likelihood ratios will be calculated using bivariate random-effects and hierarchical summary receiver operating characteristic models. In the presence of between-study heterogeneity, we will conduct stratified meta-analyses and meta-regression to determine whether diagnostic accuracy estimates vary by country of origin, use of ultrasound guidance during FNAB, qualifications of the individuals performing/interpreting FNAB, adherence to the Bethesda criteria for cytology classification, length of clinical follow-up, timing of data collection, patient selection methods, and presence of verification bias.

**Discussion:**

This meta-analysis will determine the diagnostic accuracy of FNAB for detection of malignancy in pediatric thyroid nodules and explore whether heterogeneity observed across studies may be explained by variations in patient population, FNAB technique or interpretation, and/or study-level risks of bias. This will be the first study to determine the accuracy of Bethesda cytological classification levels of FNAB (benign, atypical, follicular, suspicious, malignant). We expect that our results will help in guiding clinical decision-making in children with thyroid nodules.

**Systematic review registration:**

PROSPERO No. CRD42014007140

## Background

Thyroid nodules are uncommon in children, with a prevalence ranging from 0.05 to 2% [1–6]. Nodules are more likely to be found in girls than boys and in adolescents compared to their younger counterparts [7, 8]. Although nodules have a low risk of malignant transformation in adults (5 to 15%), the incidence in pediatric patients is estimated to be as high as 70% [2, 5, 7, 9]. Risk factors for thyroid malignancy in children include family history of thyroid cancer, certain genetic mutations, and exposure to therapeutic or environmental irradiation.

Some authors have advocated that the increased malignant potential of thyroid nodules in children justifies the liberal use of surgical exploration in several pediatric populations [5]. However, although thyroid surgery is typically well-tolerated, the potential for associated complications deters many clinicians from proceeding directly to operation [10–12]. Risks of thyroid surgery include hypothyroidism, hypoparathyroidism, recurrent laryngeal nerve injury, and postoperative bleeding and infection. These risks increase during completion thyroidectomy if a malignancy is found after hemithyroidectomy [13]. Thus, an accurate diagnostic test is essential to facilitate pre-operative decisions regarding management of pediatric thyroid nodules.

Fine needle aspiration biopsy (FNAB), also known as fine needle aspiration cytology, has been used since the early 1980s to classify the cytology of (and thereby diagnose) suspicious superficial soft tissue lesions. Improvements in ultrasound (US) technology have led to increased detection of incidental thyroid nodules and, consequently, more frequent use of FNAB [14]. A generalist (family practitioner, pediatrician, or internist) or a specialist (endocrinologist, surgeon, radiologist, or pathologist) may perform this procedure, with or without US guidance (which, in theory, may lead to heightened accuracy and increased safety). As comfort levels with FNAB have increased, greater confidence in the accuracy of cytology results has reduced the number of thyroid surgeries for benign nodules [15–17]. However, most diagnostic accuracy studies of FNAB for prediction of malignancy in thyroid nodules have focused on adult subjects, leading pediatric clinicians to question whether its reported accuracy is generalizable to children [18–20].

In 2007, the Thyroid FNAB State of the Science Conference addressed the varying terminology in FNAB reporting, concluding that inconsistencies prevented comparisons of diagnoses across different sites. Prior to the conference, most pathologists classified FNAB cytology as inadequate, benign, malignant, or indeterminate using variable definitions. Discussions at this conference resulted in the publication of the Bethesda System for Reporting Thyroid Cytopathology (also known as the Bethesda criteria) in 2009. The Bethesda criteria classify FNAB samples as non-diagnostic, benign, atypia/follicular lesion of undetermined significance, follicular neoplasm or suspicious for follicular neoplasm, suspicious for malignancy, or malignant [21]. The largest benefit of these criteria is that they clearly describe and link each of these categories to a risk of malignancy, facilitating prognostication and clinical decision-making regarding surgery or non-operative/conservative management [22]. After introduction of this classification scheme, the American Thyroid Association endorsed FNAB as the standard of care in North America for evaluation of thyroid nodules in their clinical practice guidelines [23].

Although a meta-analysis was published in 2009 evaluating the accuracy of FNAB for detection of malignancy in pediatric thyroid nodules, another systematic review is urgently required for several reasons [24]. First, multiple relevant articles have been published since the last review by Stevens et al*.* [24], potentially altering conclusions of the study. Second, their meta-analysis reviewed literature published prior to January 2007 (that is, before introduction of the Bethesda criteria) and included minimal data on the use of US guidance during FNAB. Third, Stevens et al. [24] did not directly address the risk of design-related biases among the included articles—biases that have previously been shown to overestimate the reported accuracy of a diagnostic test—potentially limiting or even preventing clinical application of their findings [24–26]. In particular, as clinicians may elect to follow patients clinically rather than proceed to thyroid surgery after a non-malignant FNAB result, this will prevent comparison against the gold standard of surgical histopathology. Thus, partial verification bias is expected to be a major limiting factor in pediatric FNAB diagnostic accuracy studies. As the previous study did not assess these potential sources of bias and heterogeneity, an updated and more elaborate systematic review and meta-analysis could verify or potentially refute the applicability of their findings to current pediatric clinical practices. The objective of this study is to systematically review the diagnostic accuracy of FNAB for the detection of thyroid malignancy.

## Methods

### Protocol

This study adopts recommendations on the conduct and reporting of systematic reviews and meta-analyses outlined by the Preferred Reporting Items in Systematic Reviews and Meta-Analyses statement, the Meta-Analysis of Observational Studies in Epidemiology proposal, and the Cochrane Diagnostic Test Accuracy Working Group [27–30]. The protocol is registered in the PROSPERO International Prospective Register of Systematic Reviews (Registration No. CRD42014007140).

### Focused clinical question

In pediatric patients with a thyroid nodule, is FNAB as accurate as surgical histopathology or clinical follow-up for the detection of thyroid malignancy?

PICOD components

- Population
  - Patients ≤18 years of age, or those defined as exclusively pediatric patients by the authors, with a thyroid nodule that is palpable or seen on diagnostic imaging
- Intervention
  - FNAB of the thyroid nodule, with or without US guidance
- Comparison
  - Surgical histopathology or clinical follow-up
- Outcome
  - Test accuracy for detection of thyroid malignancy as defined by the authors, including true and false positives and negatives, sensitivity and specificity, and positive and negative likelihood ratios
- Design
  - Diagnostic accuracy studies [30]

Primary outcome

- Test accuracy of FNAB for as defined by the authors

Secondary outcomes

- Test accuracy of FNAB for classification of lesions according to the Bethesda criteria (non-diagnostic, benign, atypia/follicular lesion of undetermined significance, follicular neoplasm or suspicious for follicular neoplasm, suspicious for malignancy, malignant). This outcome was chosen as secondary instead of primary to allow for a comprehensive evaluation of the accuracy of FNAB for classifying thyroid nodules in children (according to both Bethesda and non-Bethesda criteria)
- Test accuracy of FNAB with or without US guidance for detection of thyroid malignancy

### Search strategy

We will search Ovid MEDLINE and EMBASE, the Cochrane Database of Systematic Reviews, and Evidence-Based Medicine from their date of first inception, without language, publication date, or other restrictions. PubMed will also be searched to capture articles not yet indexed in MEDLINE. We will also use the PubMed “related articles” feature for articles included in the systematic review and manually search the table of contents for the *Journal of Pediatric Surgery* from January 2007 onward. To identify unpublished and/or ongoing studies, we will contact experts in the field and search clinical trials registries (ClinicalTrials.gov and Current Controlled Trials), reference lists of included articles, and conference proceedings of major pediatric surgery (American Pediatric Surgical Association, Canadian Association of Pediatric Surgeons, and Pacific Association of Pediatric Surgeons) and pediatric endocrinology (European Society for Pediatric Endocrinology and Pediatric Endocrine Society/Lawson Wilkins Pediatric Endocrine Society) meetings from 2007 to 2015.

With the assistance of an information scientist/medical librarian, we developed search filters encompassing the themes thyroid, biopsy, and pediatrics, using a combination of keywords and Medical Subject Heading (MeSH)/Emtree terms (Table 1). These three themes will be combined in MEDLINE and EMBASE using the Boolean operator “AND.” A diagnostic accuracy theme will not be used as it has been shown to potentially lead to the exclusion of relevant articles in systematic reviews of diagnostic accuracy studies [30–32]. A similar search strategy using themes and Boolean operators will be performed in remaining databases.

**Table 1:** Electronic database search strategies

	Ovid MEDLINE, Cochrane Database of Systematic Reviews, PubMed, Evidence-Based Medicine reviews		Ovid EMBASE	
Search filter	MeSH terms	Text words	Emtree terms	Text words
Thyroid	Thyroid gland, thyroid nodule, thyroid neoplasm,	Thyroid*	Thyroid gland, thyroid nodule, thyroid tumor, thyroid carcinoma, thyroid cancer	Thyroid*
Biopsy	Biopsy; biopsy, fine-needle; biopsy, needle; image-guided biopsy; biopsy, large-core needle;	FNA, core needle biopsy, biopsy*, aspirat*, core, needle, cytology	Needle biopsy, biopsy, image guided biopsy, biopsy needle, large core needle biopsy, tumor biopsy, biopsy technique, fine biopsy needle, core biopsy needle, fine needle aspiration biopsy	Fna, core needle biopsy, biops* aspirat*, core, needle, cytology
Pediatric	Pediatrics, adolescent, child	Adol*, child*, ped*, paed*	Pediatrics, adolescent, child	Adol*, child* ped* paed*

*MeSH* medical subject headings

### Inclusion and exclusion criteria

After removing duplicate citations, two investigators (SWL, KYW) will independently screen all remaining titles and abstracts in duplicate. This initial screen will be broad intentionally to avoid missing potentially relevant citations. We will subsequently review the full text of any citations that appear to satisfy the following criteria:

- Patients ≤18 years of age or described to be pediatric by the author(s)
- FNAB performed on the thyroid

Those articles identified for full text review will subsequently be read independently in full by the same two investigators (SWL, KYW) to determine their eligibility for inclusion in the systematic review. We will use the following inclusion/exclusion criteria based on PICOD:

Inclusion criteria

**Fig. 1:**
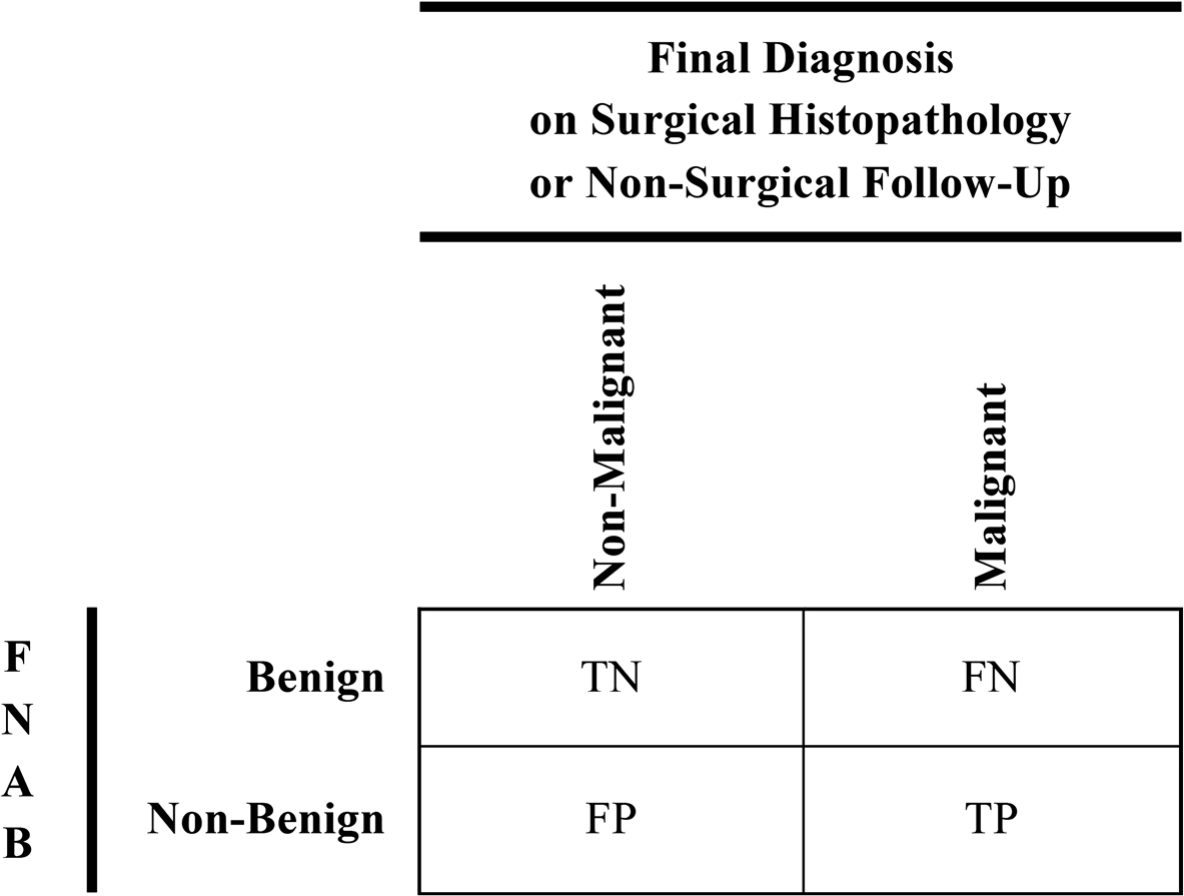
Two-by-two table examining the primary outcome. Definitions of true and false positives and negatives comparing fine needle aspiration biopsy (FNAB) to the final diagnosis based on surgical histopathology or non-surgical clinical follow-up. Positive and negative results of index test (FNAB) separated into non-benign and benign. Positive and negative results of reference test (surgical histopathology or non-surgical clinical follow-up) separated into malignant and non-malignant. *TN* true negative, *FN* false negative, *FP* false positive, *TP* true positive, *FNAB* fine needle aspiration biopsy
- Design
  - Diagnostic accuracy studies (single gated) that compare the results of an index test to the results of a reference standard on the same subjects [33–35]

- Population
  - The study population consisted of patients ≤18 years of age (or patient populations where the study authors did not provide summary estimates describing age, but did report that the included patients were exclusively children), with a thyroid nodule that is palpable or seen on diagnostic imaging
  - Data for at least ten pediatric patients were reported (to exclude case reports and small case series)
- Intervention
  - The index test was FNAB of a thyroid nodule, with or without US guidance
- Comparison
  - The reference standard was surgical histopathology or clinical follow-up
- Outcome
  - The studies examined test accuracy for detection of thyroid malignancy as defined by the authors, including true and false positives and negatives, sensitivity and specificity, and positive and negative likelihood ratios
  - Sufficient data were presented to tabulate the results comparing FNAB to surgical pathology or clinical follow-up into two-by-two contingency tables (Fig. 1)

Exclusion criteria

- Non original data
  - Duplicate data sets
  - Overlapping data sets
    - Articles with smaller cohorts will be excluded
    - Authors will be contacted to clarify their patient population if the degree of overlap is unclear
  - Non-human studies
  - Studies involving patients with exclusively malignant or benign thyroid surgical histopathology

Two investigators (SWL, KYW) will pilot test inclusion and exclusion criteria using 20 randomly selected articles to ensure complete investigator agreement of the criteria. Agreement regarding inclusion and exclusion of full-text articles between the two investigators (SWL, KYW) will be quantified using the kappa statistic. A kappa statistic greater than 0.6 will be considered moderate agreement [36]. Disagreements will be resolved by consensus or arbitration by a third party (DJR or DMR) after the article of interest has been re-read in full by all investigators [37].

### Data extraction

**Fig. 2:**
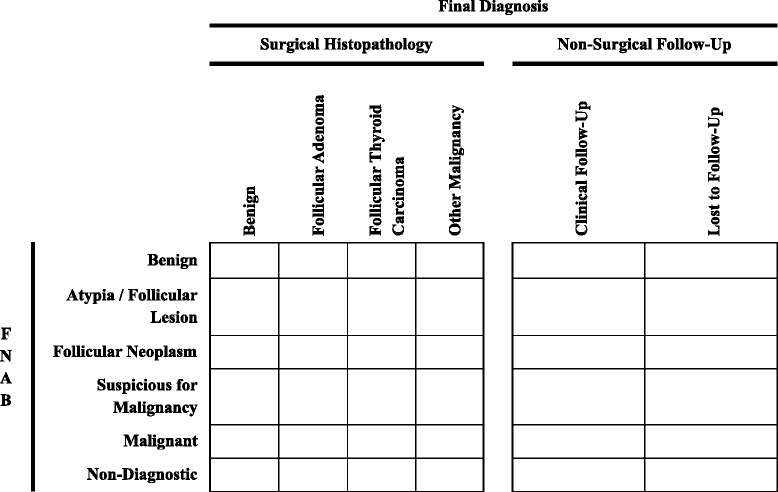
Six-by-six table examining the secondary outcome in studies that report data using the Bethesda criteria. *FNAB* fine needle aspiration biopsy

Two investigators (SWL, KYW) will extract data from all eligible diagnostic studies independently and in duplicate using a predesigned Microsoft Access 2010 (Microsoft, Redmond, WA) database form. This database form will be pilot tested on a random sample of five included studies until reliable data extraction is confirmed (kappa statistic > 0.6) [36]. We will extract the following data from included studies:

1. Study information
  First author
  Title
  Year of publication
2. Study design and methodology
  Directionality of data collection
    Retrospective, prospective
  Participant selection method
    Consecutive, random
  Inclusion and exclusion criteria
    Including whether thyroid surgery was listed as a prerequisite for enrolment
  Study setting
    Country of origin, single versus multi-site
3. Patient sample information
  Sample size
  Participant characteristics
    Age, gender
4. Experimental (index) test (FNAB)
  FNAB description
    Number of biopsies, complications, use of US guidance, qualifications of the individual performing FNAB (general practitioner, pediatrician, endocrinologist, surgeon, radiologist, pathologist)
  FNAB reporting
    Adherence to Bethesda or other criteria, qualifications of pathologist reporting results (pathologist, cytopathologist, pediatric pathologist, pediatric cytopathologist)
5. Reference standard test
  Type of surgery performed (total thyroidectomy, hemithyroidectomy, surgical biopsy)
  Length of time between FNAB and surgery
  Results of surgical histopathology (benign versus malignant, type of malignancy)
  Qualifications of pathologist reporting results (pathologist, pediatric pathologist)
  Number of patients who did not proceed from FNAB to surgery
  Length and type of follow-up (clinical, radiological)
  Number of patients lost to follow-up
6. Blinding of the pathologists to the results of FNAB and surgical histopathology
7. Study results and analysis
  Data to populate a two-by-two table (Fig. 1) to assess the primary outcome for FNAB
    Figure 1 defines true and false positives and negatives based on the two-by-two table. Positive and negative results of index test (FNAB) will be separated into benign and non-benign. Positive and negative results of gold standard reference test (surgical histopathology) and surrogate reference test (clinical follow-up) will be separated into malignant and non-malignant.
    This table will be used to generate pooled estimates of diagnostic accuracy (sensitivity, specificity, positive and negative likelihood ratios) as our primary outcome
  For our secondary outcome analysis, where possible, we will extract data to populate six-by-six tables (Fig. 2) to assess the accuracy of FNAB using the six Bethesda classifications (non-diagnostic, benign, atypia/follicular lesion of undetermined significance, follicular neoplasm or suspicious for follicular neoplasm, suspicious for malignancy, malignant), compared with six potential outcomes: four surgical (benign, follicular adenoma, follicular thyroid carcinoma, other malignancy), and two non-operative (clinical follow-up, loss to follow-up).

**Fig. 3:**
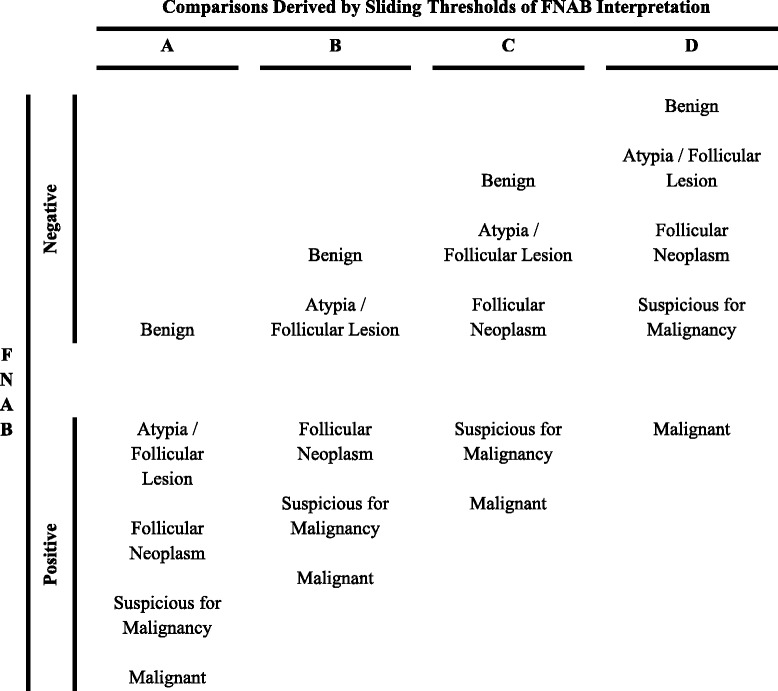
Sliding thresholds. Sliding thresholds used for fine needle aspiration biopsy (FNAB) interpretation as test negative or positive. Four separate comparisons (A, B, C, D) evaluate the test accuracy of each FNAB diagnostic category to predict thyroid nodule malignancy. *FNAB* fine needle aspiration biopsy
    Figure 4 defines true and false positives and negatives based on the sliding thresholds for all four comparisons. Positive and negative results of the gold standard reference test (surgical histopathology) will be separated into malignant and non-malignant. Positive and negative results of the surrogate reference test (clinical follow-up) will be separated into final diagnoses based on FNAB results. We will assume that non-malignant FNAB would be followed clinically and converted to surgical management if malignancy developed. Positive and negative results of patients lost to follow-up will be separated into final diagnoses based on the assumption that non-malignant FNAB would be followed clinically and that malignant FNAB lost to follow-up would subsequently be managed at a different facility

    To evaluate the test accuracy of each FNAB diagnostic category to predict malignancy, the six-by-six data will be condensed into multiple two-by-two contingency tables by altering the threshold of interpretation of FNAB results as test negative or positive. Figure 3 shows the sliding thresholds used for FNAB interpretation, stratified into four separate comparisons. All non-diagnostic biopsies will be removed from the diagnostic accuracy meta-analysis as initial and final diagnosis of malignant or non-malignant disease is unclear in patients clinically followed or lost to follow-up

**Fig. 4:**
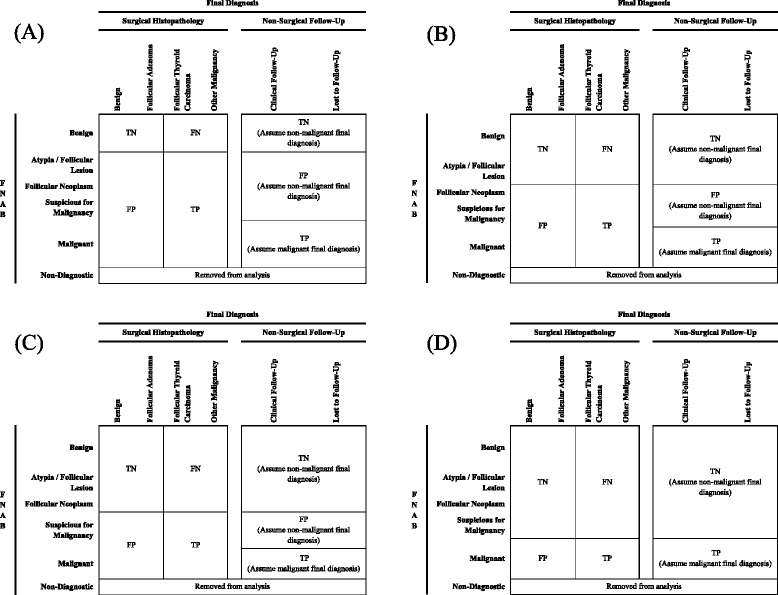
Definitions of true and false positives and negatives. Definitions of true and false positives and negatives after condensing six-by-six tables into two-by-two contingency tables for comparisons (**a**–**d**). In **a**, positive and negative results of index test (fine needle aspiration biopsy [FNAB]) separated into non-benign and benign. In **b**, positive results of FNAB including follicular neoplasm, suspicious for malignancy and malignant, and negative results of FNAB including benign and atypia/follicular lesion. In **c**, positive results of FNAB including suspicious for malignancy and malignant, and negative results of FNAB including benign, atypia/follicular lesion and follicular neoplasm. In **d**, positive results of FNAB including malignant only, and negative results of FNAB including benign, atypia/follicular lesion, follicular neoplasm and suspicious for malignancy. In all comparisons, positive and negative results of gold standard reference test (surgical histopathology) separated into malignant and non-malignant. Positive and negative results of surrogate reference test (clinical follow-up) and losses to follow-up separated into final diagnoses based on FNAB results. Non-diagnostic biopsies were removed from analysis as final diagnosis of malignant or non-malignant disease unclear in patients lost to follow-up. *TN* true negative, *FN* false negative, *FP* false positive, *TP* true positive, *FNAB* fine needle aspiration biopsy
    These tables will be used to generate multiple pooled estimates of diagnostic accuracy (sensitivity, specificity, positive and negative likelihood ratios) for each comparison

Non-English language literature will be translated by interpreters. Agreement between the two investigators (SWL, KYW) will be ensured by consensus or arbitration by a third party (DJR or DMR) as needed.

### Study quality assessment and risk of bias

The risk of bias of each article will be evaluated independently by two investigators (SWL, KYW) and reported according to the Quality Assessment of Diagnostic Accuracy Studies (QUADAS-2) tool [38]. The presence of spectrum, threshold, disease progression, and verification bias (partial or differential) will be specifically assessed, as defined below.

Spectrum bias occurs when study participants do not represent the population of interest due to inappropriate patient selection. This is an anticipated source of bias in articles where thyroid surgery forms part of the inclusion criteria. These exclusively surgical cohorts likely represent a distinct subset of the population with more worrisome findings and a higher pre-test probability for malignancy, leading to potential differences in diagnostic accuracy results. Another example of spectrum bias includes studies targeting hypothyroid or hyperthyroid patients specifically, where extrapolation to the general pediatric population with thyroid nodules may be inappropriate.

Threshold bias develops when pathologists use varying definitions to report FNAB results. This leads to a greater likelihood to diagnose benign or malignant disease based on an individual pathologist’s threshold of concern. The Bethesda criteria were introduced to standardize FNAB classification and minimize threshold bias. Studies reporting results by Bethesda versus other criteria will be compared to evaluate the potential contribution of threshold bias to diagnostic accuracy.

Disease progression bias is a concern when the interval between the index test and reference standard is long enough to potentially allow progression of disease from benign to malignant or from one type of malignant disease to another. An index test may be negative and the reference test positive due to rapid development of malignancy, rather than signify an inaccurate index test. To assess the risk of disease progression bias, an appropriate time frame between FNAB and surgery and length of clinical follow-up would need to be defined. However, this interval is not well described in the literature as the latency period for development of thyroid malignancy after discovery of a nodule may extend for years, despite exposure to known risk factors [39, 40]. As such, we will collect data regarding these parameters without imposing predefined intervals such that studies may later be categorized into those with shorter versus longer intervals for stratified meta-regression.

Partial verification bias occurs when results of the index test influence whether or not the patient receives the reference standard. There is significant potential for this type of bias among the studies that will be included in this systematic review as benign cytology may decrease the likelihood of any type of follow-up, whether surgical or clinical, unless there are other significant risk factors for malignancy. Partial verification bias frequently leads to inflated diagnostic accuracy as benign FNAB results may be assumed inappropriately to represent true negative disease [41]. Differential verification bias arises when results of the index test determine which reference standard is used to confirm the diagnosis. Using clinical follow-up as a surrogate reference standard, many studies will be prone to differential verification bias with benign cytology followed clinically instead of with surgery. Verification bias, whether partial or differential, is expected to be the primary limiting factor affecting the validity of pooled estimates across the diagnostic accuracy studies that will be included in this systematic review. In order to eliminate verification bias, in an ideal diagnostic accuracy study, all patients presenting with a nodule must undergo both FNAB and surgical excision to definitively diagnosis benign or malignant disease. However, this practice does not occur as most low-risk patients are observed in follow-up to avoid the risks of surgery. Ethically, inclusion of patients with FNAB who undergo surgery and lifelong clinical follow-up provides the best case scenario for confirming diagnostic accuracy. Verification bias may be reduced, but not eliminated, with serial clinical and radiological examinations for several years to capture any false negative FNAB, though the required duration of follow-up is unclear. It is anticipated that this systematic review will find a mixture of studies with different biases. The interconnectedness of spectrum and verification bias in this setting will also be assessed, since studies with surgical cohorts prone to spectrum bias are also at low risk of verification bias (i.e., all patients will have definitive surgical histopathology).

As a supplement to the QUADAS-2 tool, we will also examine the timing of data collection (prospective, retrospective), the qualifications of the individual performing the FNAB (general practitioner, pediatrician, endocrinologist, surgeon, radiologist, pathologist) or the interpreting pathologist (general pathologist, cytopathologist, pediatric pathologist, pediatric cytopathologist), and adherence of cytology reporting to the Bethesda versus other criteria.

Disagreements between the two investigators (SWL, KYW) will be resolved by consensus or arbitration by a third party (DJR or DMR).

### Data synthesis and analysis

True and false positives and negatives will be defined by two-by-two contingency tables (Fig. 1) for the primary outcome. True and false positives and negatives will be defined after condensing six-by-six tables (Fig. 2) into two-by-two contingency tables (Fig. 4) for the secondary outcome that will examine each Bethesda classification level. These tables will be used to calculate study-level estimates of sensitivity, specificity, and positive and negative likelihood ratios for detection of thyroid malignancy. Hierarchical summary receiver operating characteristic (HSROC) curves will be generated to depict the bivariate relationship between individual study estimates of sensitivity and specificity [30, 42, 43]. We will also use this model to calculate the proportion of between-study heterogeneity that may be due to diagnostic threshold variability using the between-study covariance parameter [30, 43–45].

Bivariate random-effects models will be used to derive pooled estimates of sensitivity, specificity, and positive and negative likelihood ratios for detection of malignancy with FNAB [43, 45, 46]. These models incorporate the degree of negative correlation that may exist between sensitivity and specificity across studies [43, 45, 46]. This joint synthesis of diagnostic accuracy estimates is unbiased despite diagnostic threshold variability and facilitates the development of Bayesian probability modifying and Fagan plots [42, 43, 45–47]. These two plots will allow for an assessment of the likely post-test probability obtained after applying FNAB to samples of patients with varying ranges of pre-test probabilities of thyroid malignancy. These models will also allow us to determine the extent of heterogeneity (due to diagnostic threshold variability or study-level covariates) in our pooled estimates through the production of forest plots and the computation of *I*^2^- and *Q*-statistics [45–49].

In the presence of inter-study heterogeneity, we will use the bivariate model to conduct subgroup analyses and meta-regression to determine whether a number of pre-defined covariates may explain variation in reported diagnostic performance results across studies [43–46, 48–50]. Covariates of interest will include those describing the study setting (country of origin, single versus multi-site), risk of bias (prospective versus retrospective data collection, random versus consecutive method of selection, thyroidectomy as part of the inclusion criteria, presence of verification bias, length of follow-up, loss to follow-up greater than 15 %), FNAB implementation and interpretation (use of US guidance, qualifications of individual performing and interpreting FNAB, use of Bethesda or other criteria), and length of clinical follow-up. We will also examine whether any studies exert undue influence on our pooled diagnostic accuracy estimates by performing a sensitivity analysis, removing those that appear to be influential outliers or those which may include potentially overlapping patients. Influential studies will be identified using spike plots of Cook’s distance and scatter plots of standardized residuals [42, 43, 51–53]. Finally, to assess for the presence of small study effects potentially due to publication bias, we will create funnel plots using the diagnostic odds ratio and conduct Deek’s asymmetry tests [54].

All statistical analyses will be performed using Stata version 13.1 (Stata Corp, College Station, TX), including the “midas” and “metandi” command packages [42, 43, 55].

## Discussion

Thyroid nodules can provoke anxiety in children, families, and physicians alike due to diagnostic uncertainty in the setting of greater potential for malignancy. The ability of a diagnostic test to distinguish malignant from benign disease is paramount for clinicians to provide appropriate counselling regarding treatment and prognostication. In addition to providing a systematic review and meta-analysis of the diagnostic accuracy of FNAB in pediatric thyroid nodules for the detection of malignancy, this will be the first study to determine the accuracy of FNAB according to the Bethesda criteria. In doing this, our results may serve as a better guide for clinical decision-making in children with thyroid nodules.

Although the American Thyroid Association endorses FNAB as the standard of care in North America for the evaluation of thyroid nodules in adults and children, the evidence supporting this recommendation is likely based on the results of studies conducted among adults. Pediatric studies may be limited by several study-level biases. Thus, this systematic review and meta-analysis will rigorously examine the potential magnitude of influence that individual study-level biases may have on the diagnostic accuracy of FNAB. Other specific aims to be addressed by this study include determining the value of adherence to the Bethesda criteria, US guidance, and the qualifications of the individual performing and interpreting the FNAB on the diagnostic accuracy of FNAB. If these factors are found to enhance diagnostic accuracy, this may support the need for routine referral of children with thyroid nodules to specialty centres where US and FNAB-trained personnel are available to improve patient care and outcomes.

## Abbreviations

CI: confidence interval
FN: false negative
FP: false positive
FNAB: fine needle aspiration biopsy
HSROC: hierarchical summary receiver operating characteristic
MeSH: Medical Subject Heading
QUADAS: Quality Assessment of Diagnostic Accuracy Studies
TN: true negative
TP: true positive
US: ultrasound
